# Pharmacokinetics and pharmacodynamics of fenoldopam mesylate for blood pressure control in pediatric patients

**DOI:** 10.1186/1471-2253-8-6

**Published:** 2008-10-06

**Authors:** Gregory B Hammer, Susan T Verghese, David R Drover, Myron Yaster, Joseph R Tobin

**Affiliations:** 1 Departments of Anesthesia and Pediatrics, Stanford University School of Medicine, Stanford, USA; 2 Departments of Anesthesia and Pediatrics, Children's National Medical Center, George Washington University School of Medicine, Washington, USA; 3 Department of Anesthesia, Stanford University School of Medicine, Stanford, USA; 4 Departments of Anesthesiology, Critical Care Medicine, and Pediatrics, Johns Hopkins University, Baltimore, USA; 5 Departments of Anesthesiology and Pediatrics, Wake Forest University School of Medicine, Winston-Salem, USA

## Abstract

**Background:**

Fenoldopam mesylate, a selective dopamine1-receptor agonist, is used by intravenous infusion to treat hypertension in adults. Fenoldopam is not approved by the FDA for use in children; reports describing its use in pediatrics are limited. In a multi-institutional, placebo controlled, double-blind, multi-dose trial we determined the pharmacokinetic (PK) and pharmacodynamic (PD) characteristics and side-effect profile of fenoldopam in children.

**Methods:**

Seventy seven (77) children from 3 weeks to 12 years of age scheduled for surgery in which deliberate hypotension would be induced were enrolled. Patients were randomly assigned to one of five, blinded treatment groups (placebo or fenoldopam 0.05, 0.2, 0.8, or 3.2 mcg/kg/min iv) for a 30-minute interval after stabilization of anesthesia and placement of vascular catheters. Following the 30-minute blinded interval, investigators adjusted the fenoldopam dose to achieve a target mean arterial pressure in the open-label period until deliberate hypotension was no longer indicated (e.g., muscle-layer closure). Mean arterial pressure and heart rate were continuously monitored and were the primary endpoints.

**Results:**

Seventy-six children completed the trial. Fenoldopam at doses of 0.8 and 3.2 mcg/kg/min significantly reduced blood pressure (p < 0.05) during the blinded interval, and doses of 1.0–1.2 mcg/kg/min resulted in continued control of blood pressure during the open-label interval. Doses greater than 1.2 mcg/kg/min during the open-label period resulted in increasing heart rate without additional reduction in blood pressure. Fenoldopam was well-tolerated; side effects occurred in a minority of patients. The PK/PD relationship of fenoldopam in children was determined.

**Conclusion:**

Fenoldopam is a rapid-acting, effective agent for intravenous control of blood pressure in children. The effective dose range is significantly higher in children undergoing anesthesia and surgery (0.8–1.2 mcg/kg/min) than as labeled for adults (0.05–0.3 mcg/kg/min). The PK and side-effect profiles for children and adults are similar.

## Background

Depression of systemic arterial pressure, or deliberate hypotension, is desirable in selected patients during surgery. Deliberate hypotension helps reduce bleeding, thereby improve surgical conditions; decreases the need for blood transfusions; and may prevent organ injury caused by hypertension during periods of acute physiologic stress [1,2]. Several pharmacologic agents have been used to control blood pressure during surgery in children, including inhalation anesthetics, direct-acting vasodilators (e.g., sodium nitroprusside, nitroglycerin) [3,4], calcium channel-blockers (nicardipine) [3], and beta-adrenergic antagonists (esmolol, labetalol) [5].

Fenoldopam mesylate is a rapid-acting vasodilator used to manage hypertension in adults [6]. It is a selective dopamine_1_-receptor agonist, producing vasodilation of the renal, mesenteric, peripheral, and coronary arteries [7]. Therapeutic doses increase renal blood flow, creatinine clearance, urinary flow, and excretion of sodium. Fenoldopam has no significant affinity for dopamine_2 _receptors. In adults, steady-state plasma concentrations are achieved rapidly, and they are proportional to infusion rate. Plasma concentrations and clinical effects dissipate quickly in adult patients, once the infusion is discontinued [8]. Fenoldopam has also been shown to control blood pressure in adult patients with hypertensive emergencies and in the postoperative period.[9] However, data are limited on the use of fenoldopam in pediatric patients [10].

This study was performed to define the pharmacokinetics (PK), pharmacodynamics (PD) and side-effect profile of fenoldopam in pediatric patients undergoing surgery.

## Methods

Our study was a randomized, multicenter, placebo-controlled, dose-ranging, two-phase study. The protocol, designed with consultation from the United States Food and Drug Administration (FDA), was approved by the Institutional Review Board from each participating institution. Written informed consent, and patient assent, when applicable, were obtained.

The initial blinded, placebo-controlled, dose-ranging phase was followed by an open-label, dose-titration phase. Children up to 12 years of age and Tanner Stage 1 or 2 for whom deliberate hypotension was planned for at least 2 hours during surgery were eligible for enrollment. Surgical cases included posterior spinal fusion and other major orthopedic, craniofacial and neurosurgical procedures. Exclusion criteria included weight < 2 kg, allergy to fenoldopam or its metabolites (e.g., sulfite or metabisulfite), and contraindication for the use of dopaminergic agents (e.g., known history of glaucoma, existing or projected development of intracranial hypertension).

Patients were randomized in equal proportions to 1 of 5 blinded infusion-treatment groups: placebo or fenoldopam 0.05, 0.2, 0.8 or 3.2 mcg/kg/min, administered as a continuous iv infusion for 30 minutes. After 30 minutes of blinded study-drug administration, an open-label fenoldopam titration period was initiated until blood pressure control was achieved.

Anesthesia was induced with propofol, sevoflurane, or halothane as determined by the clinician. Muscle relaxation, if required, was achieved with either vecuronium or cisatracurium. Fentanyl was used for analgesia. The choices of drugs used for anesthetic maintenance, neuromuscular blockade, and analgesia were based on the clinical needs of the individual patient, but they were kept constant during the blinded infusion period. Blinded drug administration (or placebo) began after stabilization of anesthesia and placement of an arterial catheter for blood-pressure measurement and sampling. Vital signs, including mean arterial pressure (MAP) and heart rate (HR) were continuously monitored and recorded just prior to drug administration and every 5 minutes throughout the study. Continuous electrocardiography and pulse oximetry were also performed throughout the study.

Following 30 minutes of blinded drug or placebo administration, an open-label infusion of fenoldopam was initiated at 0.1 mcg/kg/min. The dose was then titrated based on the patient's MAP and HR response. A target MAP was chosen for each patient such that MAP did not fall below 50 mmHg (40 mmHg for neonates). Dose adjustments were made in increments of 0.3 to 0.5 mcg/kg/min at intervals of at least 20 minutes to reach the target MAP. If MAP fell below 50 mmHg (40 mmHg for neonates) or more than 20% below the target MAP, or if HR exceeded the age-adjusted maximum with no other explainable cause (e.g., concomitant medication, surgical stimulation or hemorrhage/hypovolemia), open-label fenoldopam administration was discontinued until MAP and HR returned to protocol limits. Fenoldopam was subsequently restarted at a dose 20 to 50% less than the previous dose. If discontinuation of fenoldopam for 20 minutes did not return MAP and HR to within protocol limits, the open-label study period was terminated.

A blood sample for PK analysis was collected from all patients 20 minutes after the start of blinded drug administration. In addition, for patients ≥ 2 years of age, blood samples for PK analysis were collected at 5, 10, or 15 minutes after the start of blinded study drug administration (a total of two samples per patient) according to a sparse sampling protocol. Blood samples for PK analysis were also collected from all patients immediately prior to and 5 minutes after termination of open-label fenoldopam administration. Total blood sample volumes for PK analysis were constructed by design not to exceed 1% estimated blood volume for safety, so that fewer samples for analysis were drawn from children < 2 years of age. A complete blood count and chemistry panel were performed prior to and after surgery.

### PK and PD

Plasma concentrations of fenoldopam were determined using a validated, high-performance liquid chromatography method with mass spectrometric/mass spectrometric detection (HPLC-MS/MS). The PK of fenoldopam was assessed via population compartmental modeling and via non-compartmental methods. The non-compartmental PK variables were elimination rate constant, corresponding half-life (t_1/2_), plasma concentration at 20 minutes into the blinded infusion period (C_20_), and clearance (CL). The elimination rate constant was estimated using the plasma concentrations at the end of and after open-label study drug administration, and the data were explored for a trend with age, using linear regression analysis and tests for trend. The C_20 _values were used to evaluate the linear kinetics of fenoldopam in pediatric patients. The CL values were estimated by infusion rate divided rate by C_20_. The pharmacokinetics parameters were estimated with a mixed-effects non-linear fitting approach using the program NONMEM, version V[11]

The primary PD variables were MAP and HR. NONMEM was used to model the relationship between the PD variable (either MAP or HR) and fenoldopam plasma concentrations.

### Onset and Offset of Effect

The time until onset of effect was determined to be the earliest measurement of MAP during the blinded infusion period wherein statistically significant differences between active drug and placebo data were observed. Time until offset of effect was calculated using the vital sign data collected during the 30-minute interval just after open-label study drug discontinuation. Offset was defined as the lesser of a 10 mmHg or 10% increase in MAP for at least 2 consecutive measurements. For patients who failed to satisfy the above criteria during the 30 minutes following discontinuation of the drug, the time of offset was assumed to be greater than 30 minutes.

### Efficacy

The primary efficacy variable was MAP. An analysis of covariance was performed on the change from baseline MAP (last measurement before the beginning of fenoldopam) to each scheduled measurement during the blinded infusion period. Within the framework of this analysis, each fenoldopam dose was compared to placebo. In addition, ANOVA was performed on a contrast in the treatment effects that reflected a trend with dose, with placebo considered a 0 mcg/kg/min dose.

### Side-effects

All patients administered fenoldopam were included in the side-effects analyses. The number and percentage of patients having drug-related adverse events were tabulated by the COSTART term and body system. Each treatment group was analyzed, and all groups combined were analyzed. An analysis of covariance was performed for HR change from baseline to each scheduled time of measurement during the blinded infusion period.

## Results

### Demographics

Seventy-seven (77) randomized patients were enrolled. There were 15, 15, 16, 15, and 15 patients who were administered placebo, 0.05, 0.2, 0.8, and 3.2 mcg/kg/min fenoldopam, respectively. One patient received no drug due to a pump malfunction and was excluded from the study. No statistically significant differences were observed among the treatment groups in gender, mean age, height, or weight (Table 1). The majority of patients were males (56%, 43/77) and Caucasians (65%, 50/77). The mean age was 51.9 months, with a range from 3 weeks to 12.75 years.

**Table 1:** Demographic information

		**Fenoldopam (mcg/kg/min)**				
**Demographic **	**Placebo **	0.05	0.2	0.8	3.2	P-value^a^
**Characteristic**	(N = 16)	(N = 16)	(N = 15)	(N = 15)	(N = 15)	
Gender						0.802
Female	6 (38%)	7 (47%)	8 (50%)	5 (33%)	8 (53%)	
Male	10 (63%)	8 (53%)	8 (50%)	10 (67%)	7 (47%)	
Age (months)						0.917
Mean (SD)	45.6 (48.82)	60.6 (48.82)	55.9 (50.15)	49.3 (54.92)	48.2 (45.97)	
Height (cm)	(N = 15)	(N = 13)	(N = 14)	(N = 14)	(N = 15)	0.884
Mean (SD)	84.7 (27.59)	95.1 (32.65)	91.4 (32.17)	94.8 (34.32)	89.8 (22.24)	
Weight (kg)						0.687
Mean (SD)	14.7 (9.04)	18.3 (13.55)	19.1 (14.57)	16.2 (10.95)	14.1 (7.07)	

SD = standard deviation

^a ^P-values are from a Fisher's exact test (gender and age category) or a one-way analysis of variance (age, height, weight).

### Study Discontinuations

The study drug was prematurely discontinued in four patients during the blinded period. In two of these patients receiving fenoldopam 3.2 mcg/kg/min, the drug was discontinued because of a reduction in MAP beyond the protocol specified limit (< 50 mmHg). The third patient, administered placebo, and the fourth, administered 0.2 mcg/kg/min, were discontinued because the investigator decided the study drug was ineffective at reducing their MAP to the target level. The study drug was discontinued in twenty-seven patients during the open-label period,: seven were discontinued due to adverse events (AEs) and twenty due to other reasons (e.g., hypotension no longer desired). The AEs that caused the open-label study drug to be discontinued included hypotension (N = 3), hemorrhage (N = 3) and tachycardia (N = 1).

### Efficacy

**Figure 1:**
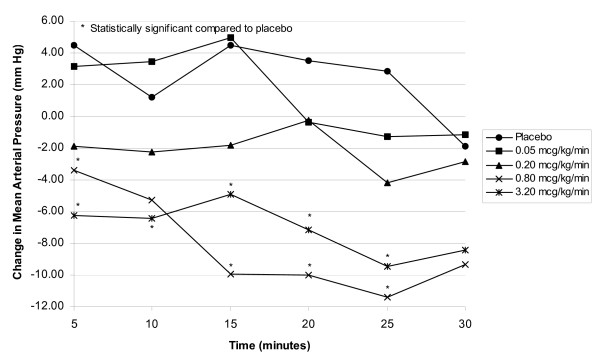
Plot of means change in MAP versus time during the blind infusion period by dose.

The results of the blinded period indicated that the lowest fenoldopam dosage that decreased MAP was 0.2 mcg/kg/min (Figure 1). The maximum MAP decrease occurred at a dosage of 0.8 mcg/kg/min. At the 0.8 mcg/kg/min dosage, statistically significant decreases in MAP compared with placebo were seen as early as 5 minutes after initiation of the fenoldopam infusion, with statistically significant hypotensive effects also present at 15, 20, and 25 minutes during the 30-minute blinded infusion period. The maximum mean decrease in MAP at the 0.8 mcg/kg/min dosage was seen 25 minutes after initiation of the infusion. Changes at the 3.2 mcg/kg/min dosage were similar to those observed at the 0.8 mcg/kg/min dosage: the maximum mean decrease was observed 25 minutes after the start of the infusion. Statistically significant decreases occurred at 5, 15, 20, and 25 minute time intervals at the 3.2 mcg/kg/min dosage. The covariate for age was not statistically significant with respect to change from baseline in MAP.

Fenoldopam dosages up to 1.2 mcg/kg/min in the open-label period caused clinically significant reductions in MAP, but dosages greater than 1.2 mcg/kg/min conveyed little additional benefit in lowering MAP. There appeared to be no loss of effect on MAP during the open-label period of the study.

### Side-effects

The average length of time that patients received fenoldopam infusion was 3.21 hours; the range was 0.50 to 6.75 hours. The average fenoldopam dose (defined as the cumulative dose divided by total amount of time of study drug infusion) was of 1.00 mcg/kg/min; the range was < 0.05 to 3.2 mcg/kg/min. The mean maximum dose was 1.90 mcg/kg/min; the range was < 0.05 to 4.10. There were no statistically significant differences in total infusion time, average dose, or maximum dose of fenoldopam.

**Figure 2:**
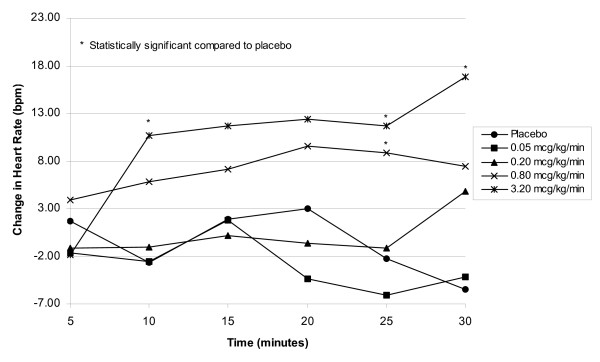
Plot of mean change in HR versus time during the blind infusion period by dose.

Compared to patients treated with placebo, patients treated with fenoldopam 3.2 mcg/kg/min experienced the greatest increases in HR at all time points except at the 5-minute time interval (Figure 2). Beginning 10 minutes after initiation of the infusion, HR values exceeded baseline at every time interval in the 3.2 mcg/kg/min dosing group. Mean increases ranged from 9 to 17 bpm, achieving statistical significance at 10, 25, and 30 minutes. Mean HR also increased at the 0.8 mcg/kg/min dosage, achieving statistical significance only at the 25-minute time interval. When the HR across each time interval was examined, the p-value for the trend indicated a statistically significant, dose-related increase at 10, 20, 25, and 30 minutes. Age was not found to be a statistically significant covariate in the analysis of change from baseline in HR.

Other than tachycardia, most AEs were considered to be unrelated to the use of fenoldopam. Four patients during the blinded period, 13 during the open-label period, and one during the follow-up period experienced one or more drug-related AEs. A summary of all AEs possibly or probably related to study drug is shown is Table 2. Hypokalemia, an AE observed with fenoldopam administration in the adult population [12], was observed in 7 of 76 pediatric patients; only one event was considered by the investigator to be possibly related to the study drug, with most cases attributed to inadequate replacement of potassium in IV fluids. No statistically significant differences were observed between any of the fenoldopam dose groups and the placebo group in mean change from baseline to final value in any hematology or chemistry parameter. Only one AE was reported during the follow-up period (oliguria).

**Table 2:** Incidence of Drug-related Adverse Event by COSTART term^a^

	Blinded Period		Open-Label Period	Follow-Up Period
	Fenoldopam	Fenoldopam		
	0.05	3.2	Fenoldopam	Fenoldopam_[PR2]_
Adverse	mcg/kg/min	mcg/kg/min	_[PR1]_(N = 74)	(N = 77_[PR3]_)
Events	(N = 15)	(N = 15)		
OVERALL	1 (7%)	3 (20%)	13 (17%)	1 (1%)
Hypotension	1 (7%)	3 (20%)	9 (12%)	0
Tachycardia	0	0	2 (3%)	0
Bradycardia	0	0	1 (1%)	0
Hemorrhage	0	0	1 (1%)	0
Hypokalemia	0	0	1 (1%)	0
Oliguria	0	0	0	1 (1%)

^a ^Adverse events considered by the investigator to be possibly or probably related to study drug.

### Pharmacokinetic/Pharmacodynamic Analyses

The pharmacokinetics of fenoldopam as assessed by the C20 values were linearly related to dose. The means ± standard errors for the elimination rate constant and CL values from the non-compartmental analyses were 12.4 ± 0.10 L/h and 3.02 ± 0.04 L/h/kg, respectively. The effect of age on the elimination rate constant was not significant.

The results of the compartmental PK analysis were consistent with the results from the non-compartmental analysis. A single-compartment model adequately described the plasma-concentration data with population central value estimates ± approximate standard errors for the elimination rate constant and CL of 13.2 ± 1.23 h^-1 ^and 3.06 ± 0.62 Lh^-1^kg^-1^, respectively. The effect of age on the elimination rate constant was not significant in the compartmental PK analysis.

The population PK/PD modeling results indicate that the relationship between fenoldopam plasma concentrations and MAP is not linear; the greatest deviation from linearity was found at the highest plasma levels. The maximum effect model was found to be the best fitting model. Average population estimates ± approximate standard errors for the maximum obtainable effect (E_max_) and the plasma concentration required to obtain half of this effect (EC_50_) were found to be -27 ± 5.4 mmHg and 9.1 ± 4.23 ng/mL, respectively. These results coincide with the observation that dosages greater than 1.2 mcg/kg/min conveyed little additional benefit with regard to lowering MAP. Covariates for both age and baseline MAP were considered in the E_max _model, but only baseline MAP was found to significantly enhance the model fit.

Population PK/PD modeling for HR indicates that plasma concentrations and HR are linearly related. The estimates ± approximate standard errors of the intercept and slope for this relationship were found to be 39 ± 6 bpm and 0.24 ± 0.05 bpm/ng/mL, respectively. Baseline HR was a significant covariate in the model, but age was not significant.

### Onset/Offset of Effect

Since statistically significant differences in change from baseline in MAP were observed between the 0.80 mcg/kg/min dose group and the placebo dose group as early as 5 minutes into the blinded infusion period, it is concluded that onset of effect is approximately 5 minutes. The median time required after discontinuation of open-label study drug to increase MAP by the lesser of 10 mmHg or 10% for at least two consecutive measurements was 19 minutes. Therefore the time-to-offset of effect is estimated to be approximately 19 minutes. Because this estimate is constrained by our requirement of 2 consecutive measurements at 5 minute intervals, it may be an overestimate.

Using the parameter estimates from the population PK/PD model fit, the onset and offset of effect were calculated to be 4.0 and 15.8 minutes, respectively.

## Discussion

Fenoldopam mesylate is a selective dopaminergic_1 _receptor agonist used as an antihypertensive drug in adult patients. Administered via continuous intravenous infusion, fenoldopam vasodilates the splanchnic and renal vasculature, decreases blood pressure, maintains or augments renal blood flow and increases creatinine clearance. There are limited published data however, describing the use of fenoldopam in children. This study was designed to evaluate the PK and PD as well as the side-effect profile of fenoldopam during deliberately induced hypotension in pediatric patients. Acknowledging earlier published experience with fenoldopam in pediatric patients, wherein effective doses were reported above the package insert label (0.05–0.3 mcg/kg/min in adults), the trial design was selected to encompass a range of 0 – 3.2 mcg/kg/min [10] The study design met FDA requirements for approval of the use of fenoldopam in children and, following completion of the trial, the FDA accepted the study data in support of its determination of effectiveness of fenoldopam and pediatric labeling.[13]

Seventy-six prepubertal children ≤ 12 years of age who required deliberately induced hypotension for at least 2 hours during surgery were studied. We found that fenoldopam 0.2 mcg/kg/min was the lowest dosage at which decreases in BP were seen, and 0.8 mcg/kg/min was the most effective dosage in achieving desired decreases in BP. No additional decrease in BP was observed as the infusion was increased to 3.2 mcg/kg/min, while heart rate was significantly elevated at this dosage. The lack of further hypotensive response at doses higher than 0.8 mcg/kg/min may be due to the reflex tachycardia induced by higher doses. Fenoldopam does not have dopamine_2 _or beta adrenergic receptor activity, but it does produce reflex-mediated increases in heart rate and cardiac index. Weber et al reported that compensatory tachycardia associated with the administration of fenoldopam and other antihypertensive agents can offset the decrease in blood pressure associated with a reduction in systemic vascular resistance that these drugs produce [7].

The maximum effects model for MAP significantly out-performed the linear model with estimates of the maximum (in magnitude) obtainable change from baseline in MAP and the plasma concentration required to obtain half this effect (27 ± 5.4 mmHg and 9.1 ± 4.23 ng/mL, respectively). Although no corresponding limit to the obtainable reduction in BP has been observed in the adult population, the higher dosages in this study far exceeded the maximum doses used to establish the efficacy and PK/PD relationship in adults [14]. It is plausible that with doses comparable to those used in this study a similar plateau effect may be detectable in adults.

The intravenous administration of fenoldopam in pediatric patients was well-tolerated. No increased incidence of any AE was noted among children treated with fenoldopam relative to adults in the relatively short duration of infusion during anesthesia. However, no behavioral or subjective complaints (e.g. headache, dizziness, or orthostatic symptoms) would have been noted in our study, because patients were under general anesthesia.

Unlike MAP, there was no identifiable upper limit to the change from baseline in HR seen in either the efficacy evaluation or the PK/PD modeling. Proportional increases in HR were seen even at the highest blinded infusion dose level of 3.2 mcg/kg/min, and the population PK/PD relationship was best described by a linear relationship between plasma concentration and change from baseline in HR. The population estimates ± approximate standard errors for the added elevation of HR (bpm) for each additional unit (ng/mL) of fenoldopam mesylate found in plasma are 0.261 ± 0.10 and 0.501 ± 0.11 for pediatrics and adults, respectively. These estimates are very similar relative to their respective variances.

Both the compartmental population estimates and non-compartmental estimates of the elimination rate constant, corresponding half-life, and clearance for pediatric patients were consistent with estimates in their well-established adult counterparts. The pediatric elimination rate constant estimates from compartmental modeling and non-compartmental methods were 13.2 ± 1.23 h-1 and 12.4 ± 0.10 h-1, respectively. These values correspond to half-lives of 3.15 minutes for the compartmental estimate and 3.35 minutes for the non-compartmental estimate. Weight-adjusted clearance values from the compartmental modeling and non-compartmental methods were 3.06 ± 0.62 L/h/kg and 3.02 ± 0.04 L/h/kg, respectively. These values are remarkably close to the adult values for the elimination rate constant (9.05 ± 1.21 h-1) and CL (1.66 ± 0.42 L/h/kg) relative to their respective variances. On the basis of the central value estimate of CL (3.06 L/h/kg), approximately 0.5 mcg/kg/min of fenoldopam would have to be infused to a pediatric patient to achieve 10 ng/mL steady-state concentration of fenoldopam. From the PK/PD model results, this plasma concentration is only slightly larger than the level (9.1 ng/mL) required to attain half the maximum (in magnitude) BP control (approximately a -13.5 mmHg reduction from baseline in MAP). This plasma concentration is also associated with an increase from baseline in HR of approximately 5 bpm based upon the PK/PD model results.

The effect of age was not significant for any of the PK, PD or side-effect profile evaluations. The dose range studied demonstrates that although fenoldopam is highly effective in children, there is no additional benefit with respect to blood pressure control with doses greater than 1.2 mcg/kg/min.

A variety of pharmacologic agents have been used to induce controlled hypotension in pediatric patients, including inhalation anesthetics, remifentanil, vasodilators, calcium channel-blockers, and beta-adrenergic antagonists. Advantages of inhalation anesthetics include their immediate availability in the operating room, ease of administration, and familiarity among anesthesiologists [15,16]. Disadvantages include myocardial depression, interference with evoked potential monitoring, and delayed offset following prolonged administration. Remifentanilhas been used for controlled hypotension in children, but no dose-response data are available [17]. Sodium nitroprusside, a potent arterial vasodilator commonly used in children, has a rapid onset and offset [3]. Its disadvantages include potential cyanide and thiocyanate toxicity, photodegradation, reflex tachycardia, impaired cerebral autoregulation, and rebound hypertension [18]. In a comparative study in adults, hypotension necessitated treatment withdrawal in a greater proportion of patients treated with sodium nitroprusside than fenoldopam [19]. Nitroglycerin is a direct-acting vasodilator that acts primarily on venous and secondarily on arterial smooth muscle. Toxicity associated with nitroglycerin is rare, although tachyphylaxis has been shown [20]. Nitroglycerin is less effective than nitroprusside for producing hypotension in children [4]. Nicardipine, a calcium channel antagonist, has an intermediate onset and duration of effect on blood pressure [16]. Its disadvantages include its long elimination half-life precluding rapid titration, reflex tachycardia, its interaction with inhalation anesthetic agents, and its potentiation of neuromuscular blockade [21,22]. In a study of adult patients with hypertension following CABG surgery, fenoldopam was found to be significantly more effective than nicardipine in decreasing mean arterial pressure [23]. Unlike sodium nitroprusside, nitroglycerin, and nicardipine, fenoldopam has been found to be regionally specific and does not elicit cerebral vasodilation [24]. This profile may be advantageous in situations where cerebral vasodilation is undesirable. Esmolol, a beta-adrenergic antagonist with rapid onset and short duration of action decreases myocardial oxygen consumption and helps control coexisting tachycardia, but it may cause excessive impairment of cardiac output in infants and must be used with caution in patients with a history of bronchospasm [25].

There are several limitations to this study. First, the anesthetic technique was not standardized due to the variety of surgical procedures performed and the number of institutions involved in the study. Second, newborns and adolescents beyond Tanner Stage II maturity were excluded. Although it may be assumed that adolescents respond to a novel agent as do adults, this assumption has not been sufficiently studied to provide specific recommended dosing ranges in this population. Third, although we observed no tolerance or tachyphylaxis to fenoldopam in this study, it remains unknown whether longer-term administration will result in tolerance or rebound hypertension. If longer-term control of blood pressure is required, it would be reasonable to initiate a longer acting agent while reducing the dose of fenoldopam until use of long-term infusions has been studied. Fourth, pediatric patients with chronic hypertension, many of whom may be receiving concomitant antihypertensive agents, were not studied. Fifth, children with significant hepatic or renal dysfunction were not studied. Finally, physiologic endpoints were limited to blood pressure and heart rate, and we did not assess regional alterations in blood flow. Whether any specific regional tissue was rendered ischemic (from limited blood flow secondary to lowered blood pressure) or edematous (from excessive blood flow secondary to regional vasodilation) was not determined.

## Conclusion

In summary, fenoldopam is a unique vasodilator with effective antihypertensive activity in children. We have described the pharmacokinetics and pharmacodynamics of fenoldopam in the pediatric population. For pediatric patients it is estimated that the time of onset of effect for fenoldopam is between 4 and 5 minutes after initiation of IV infusion and that the time of offset of effect is between 16 and 19 minutes after termination of IV infusion. The effective range, when given with anesthesia, was between 0.2 and 0.8 mcg/kg/min, with administration of higher doses resulting in tachycardia without a further reduction in MAP. No tachyphylaxis was seen in this study. Experience with longer infusions will be necessary to determine if tolerance develops.

## Abbreviations

MAP: mean arterial blood pressure; AE: adverse event; bpm: beats per minute.

## Competing interests

The authors declare that they have no competing interests.

## Authors' contributions

GBH, STV, DRD, MY, JRT have made substantial contributions to conception and design, or acquisition of data, or analysis and interpretation of data. GBH, STV, DRD, MY, JRT have been involved in drafting the manuscript or revising it critically for important intellectual content. All authors have given final approval of the version to be published.

## Pre-publication history

The pre-publication history for this paper can be accessed here:


